# Thermal imaging potential and limitations to predict healing of venous leg ulcers

**DOI:** 10.1038/s41598-021-92828-2

**Published:** 2021-06-24

**Authors:** Mahta Monshipouri, Behzad Aliahmad, Rajna Ogrin, Kylie Elder, Jacinta Anderson, Barbara Polus, Dinesh Kumar

**Affiliations:** 1 Biosignals for Affordable Healthcare, RMIT University, 124 Latrobe Street, Melbourne, VIC 3000 Australia; 2 Bolton Clarke Research Institute, Bentleigh, VIC Australia; 3 Department of Business Strategy & Innovation, Griffith University, Brisbane, QLD Australia; 4 Bolton Clarke, 31 Janefield Drive, Bundoora, VIC Australia

**Keywords:** Predictive markers, Biomedical engineering

## Abstract

Area analysis of thermal images can detect delayed healing in diabetes foot ulcers, but not venous leg ulcers (VLU) assessed in the home environment. This study proposes using textural analysis of thermal images to predict the healing trajectory of venous leg ulcers assessed in home settings. Participants with VLU were followed over twelve weeks. Digital images, thermal images and planimetry of wound tracings of the ulcers of 60 older participants was recorded in their homes by nurses. Participants were labelled as healed or unhealed based on status of the wound at the 12th week follow up. The weekly change in textural features was computed and the first two principal components were obtained. 60 participants (aged 80.53 ± 11.94 years) with 72 wounds (mean area 21.32 ± 51.28cm^2^) were included in the study. The first PCA of the change in textural features in week 2 with respect to week 0 were statistically significant for differentiating between healed and unhealed cases. Textural analysis of thermal images is an effective method to predict in week 2 which venous leg ulcers will not heal by week 12 among older people whose wounds are being managed in their homes.

## Introduction

A venous leg ulcer (VLU) is a chronic wound that occurs in 1 to 2% of the population^1–3^ with an increased likelihood of occurrence of up to 3% in individuals aged over 65^4,5^. The most common aetiology of leg ulcers is venous disease^6–9^. When diagnosed and managed appropriately, approximately 70% of VLUs can heal in 24 weeks^10,11^. The normal course of healing constitutes a reduction in wound size of 50% within four weeks^12,13^. Despite best practice management, over 30% of ulcers do not heal in the expected trajectory and may require additional interventions to improve outcomes^10,11,14,15^. This leads to considerable negative impacts on quality of life^16^ and increased costs to both the individual and the health system^3,17^ .

Currently, there is no test available that can reliably predict if a VLU will heal at the expected rate, have a delayed healing trajectory, or remain unhealed. VLU guidelines state that if the wound area hasn’t reduced by at least 20–40% after four weeks, then additional interventions are warranted^2,12,18,19^. However, this delays the instigation of an adjuvant intervention to routine care which could aid in the wound’s ability to heal in a timely manner^20^. Current assessment methods to monitor wound progress over these four weeks includes regular wound tracing and measurement including the use of digital planimetry, observation and documentation of the wound tissue type, wound edge characteristics, peri wound and surrounding skin and level and type of exudate^2^. These methods require at least weekly visits with the person living with the wound and physical contact with the wound.

Regular wound photography may be used in combination with the above methods using a readily available digital camera^21^. This method of photography uses standard three colour channels of Red, Green and Blue, known as RGB images. Whilst this is useful to visualise wound appearance at a point in time that is available for all healthcare providers in the care team, it cannot be used for accurate measurement of the changes in wound size / area and other physiological parameters over time, which are the key factors associated with healing trajectory. This is because there are large variations between images due to changes in the distance between the camera and the wound which causes scale variance, altered ambient lighting conditions, image quality and differences in camera angle across specific points in time when the image is taken^22^.

Thermal imaging has been used in several medical applications, including prediction of ulceration in the feet of people with diabetes^23^. This is because it can detect temperature differences and quantify sensitive changes in skin temperature which occur with pathological changes such as soft tissue inflammation of the skin, subsequent breakdown, and infection of ulcers^24–26^. The presence of higher local temperatures can be related to inflammation or infection, while the presence of lower temperatures can indicate a slow healing rate, mainly due to decreased oxygen in that region^27^. Thermal imaging methods have also been used in individuals at risk of developing wounds by comparing the skin temperature distribution of both feet of participants, termed as asymmetry analysis. The foot with the higher temperature is considered to be at risk of ulceration^4,23^. Using an edge detection method of thermal images, healing of diabetes related foot ulcers could be predicted^28^. However, when we used this latter method to predict healing of VLUs managed in the home setting, results were negative^29^. Given many wounds are managed in settings in which ambient and individual factors cannot be controlled, for example GP clinics and home care^15,30^, alternative methods that negate the need for such controls are necessary.

To overcome the above limitations, an alternative analysis to using edge detection of thermal imaging was considered. It is well recognised that the texture of a wound is an indicator of its ability to heal^31^. Textural analysis of thermal images provides information on spatial heat distribution when applied on thermal images. It has been hypothesized that texture analysis of VLU thermal images may predict the likelihood of ulcer healing, as there is a significant change in the texture of the wounds over the healing trajectory^31^. Therefore, this study investigated the use of texture in thermal images of VLUs compared to conventional digital planimetry, and whether it could be used to predict the healing trajectory of wounds managed in the home setting to identify the ulcers that would remain unhealed after 12 weeks. This is the first report on the usefulness of this technique in the prediction of the healing status of VLUs.

## Results

### Participants and their characteristics

Sixty individuals (mean age 80.53 ± 11.94 years) with 72 wounds participated in this study. Of the 72 wounds included in the full analysis, 17 wounds healed at 12 weeks and 55 remained unhealed. Characteristics of participants is shown in Table 1 below.

**Table 1:** Characteristic of participants.

Participant characteristics	Healed in week 12	Unhealed in week 12	Overall
Total number of participants	14	46	60
Total number of VLUs	17	55	72
Age (years ± SD)	82.42 ± 8.50	79.95 ± 12.83	80.53 ± 11.94
Female gender (n, %)	7, 50%	28, 60.8%	35, 58.3%
Duration of ulcer (weeks ± SD)	17.85 ± 10.61	134.21 ± 222.88	107.06 ± 200.94
Area of ulcer (cm^2^)	18.60 ± 62.16	22.15 ± 48.25	21.32 ± 51.28

Table 2 shows the ratio of the area of the wound for week 1/ week 0, week 2/ week 1 and week 2/ week 0 obtained using digital planimetry. The statistical analysis shows that there is significant difference between the healed and unhealed when comparing the weeks 0 with week 1 and 2. The analysis confirms that digital planimetry is suitable for detecting changes in wound area over a 3-week period.

**Table 2:** Kruskal–Wallis test—comparison between ratio of the ulcer areas across three weeks and the healing status at week 12.

Ratio of wound area	Healed			Unhealed			Normality test	Kruskal–Wallis test
	Median	Mean	SD*	Median	Mean	SD	*P* Value	*P* Value
Week1/Week0	0.68	0.75	0.36	0.91	1.07	0.75	< 0.005	0.019
Week2/Week1	0.82	0.84	0.62	0.94	0.92	0.36	< 0.005	0.168
Week2/Week0	0.50	0.66	0.77	0.88	1.08	1.50	< 0.005	0.006

*Standard deviation from the mean.

Table 3 shows the values for the ratio of area of the wound obtained using thermal images for week 1/ week 0, week 2/ week1 and week 2/ week 0, the details of which are shown in Table 3. It is seen that there is no significant difference between the healed and unhealed groups.

**Table 3:** One way ANOVA—comparison between ratio of the ulcer areas of thermal images across three weeks and the healing status at week 12.

Ratio of wound area	Healed			Unhealed			Normality test	ANOVA
	Median	Mean	SD*	Median	Mean	SD	*P* Value	*P* Value
Week1/Week0	0.97	0.98	0.04	0.99	1.00	0.03	> 0.20	0.110
Week2/Week1	1.00	1.01	0.05	0.99	0.99	0.03	> 0.20	0.123
Week2/Week0	1.01	1.00	0.05	0.88	0.99	0.03	> 0.20	0.873

*Standard deviation from the mean.

Table 4 shows results of the statistical analysis of PCA1 obtained from texture analysis of the thermal images of the wounds. The results show that there was a significant difference between the healed and unhealed ulcers for the rate of change in textural features from week 0 (base line) to week 2 (week2/ week0). The other ratios (week1/week0, and week2/week1) were not found to be statistically significant and have not been tabulated. As the PCA of the texture analysis do not directly provide any physiological information, their actual values have not been provided in the table.

**Table 4:** Analysis of variance (ANOVA) to test relationship between rate of change in textural features in week 2 with respect to the baseline (i.e. week 0) when transformed in PCA domain (week2/ week0).

Healed mean	Unhealed mean	F value	95% CI* (Tukey simultaneous CI—healed subtracted)	*p* Value (α = 0.05)
-1.57	0.48	6.23	0.414–3.703	0.015

* CI: Confidence interval.

**Figure 1:**
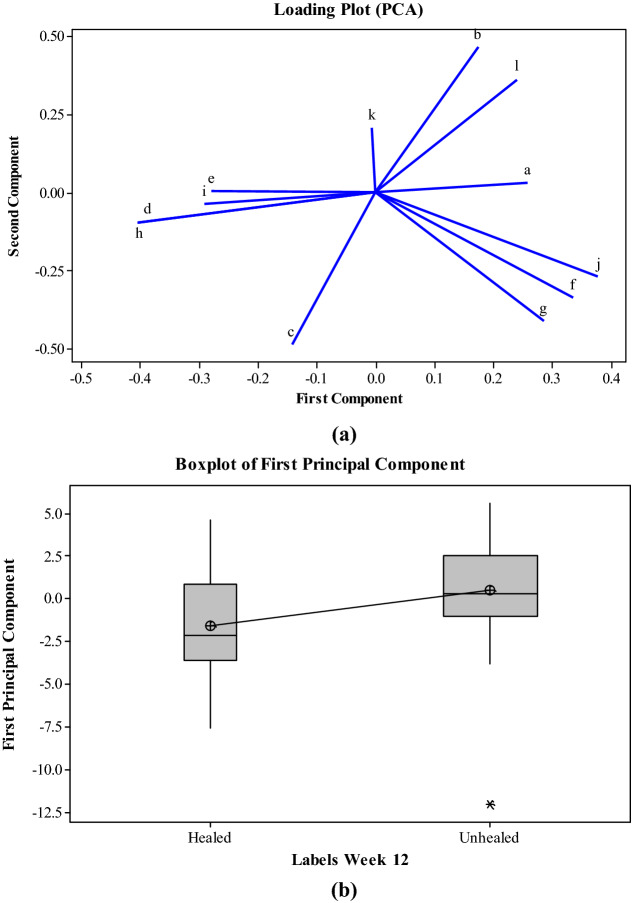
(**a**) loading plot of PCA, the letters/notations shown for each variable correspond to textural features explained in section “pre-processing and texture analysis” under item 7 (**b**) Box plot of first principal component.

Figure 1(a) shows the loading plot that graphs coefficients of each variable (i.e. textural features) for the first principal component versus the coefficients for the second principal component to identify which variable has the largest effect on each component. The notations used for each variable correspond to textural features explained in section “pre-processing and texture analysis” under item 7. According to the loading plot j, f and g (i.e. inertia, sum of square variance and sum average) have a large positive loading on the first principal component and d and h (i.e. entropy and sum entropy) have a large negative loading on the first principal component. Other variables/textural features were found to have a weak influence on the first principal component.

Figure 1(b) shows the box plot of the first principal component for healed and unhealed cases in order to better visualize how the two clusters have been separated. The second principal component was discarded as it did not show a significant difference between the healed and unhealed clusters.

## Conclusion

Digital planimetry, which is the current gold standard for predicting healing of VLUs, matches the wound healing observations over the twelve-week period used in this study. This study therefore confirms the use of this method and that digital planimetry may be used as a tool to demonstrate healing as early as two weeks after commencement of treatment. Conventional thermal imaging methods used to compute area are not suitable to monitor progress of healing when used in conditions such as in the homes of individuals with wounds due to large differences in ambient conditions and due to scaling and rotational variances. Textural analysis of thermal images is suitable for identifying unhealed wounds two weeks post intervention and is suitable for in-house observations of individuals with wounds. This latter method is resilient to natural variations in ambient conditions, scaling and rotation, and does not require contact with the wound of the individual. Furthermore, computerised analysis of textural features is a time efficient and cost-effective method to identify delayed healing of VLUs and further research to assess generalisability and to refine the methods is warranted.

## Discussion

This is the first study of texture analysis that has been undertaken on thermal images of VLUs. The rate of change in textural features between week 0 (baseline) and week 2 (third week) were significantly different for the healed and unhealed cases. Therefore, this method of thermal imaging analysis can predict whether the VLU would heal in 12 weeks by week 2 from the baseline assessment. This is an improvement on the current guidance using digital imaging or planimetry wound tracings to detect the healing wounds by week four^18^.

The significance of this work is that there is now a method for detecting those wounds that do not heal in the normal trajectory by week 2 using a non-contact, quick, objective and simple method. While digital planimetry of wound area can be used to predict healing trajectory at two weeks from baseline^32^, confirmed by this work, digital planimetry requires physical contact with wounds. A non-contact method, like thermal imaging, would be ideal to use when managing wounds in the home setting to minimise physical contact, and thereby reduce infection risk.

Textural analysis of thermal images has fewer limitations when compared to other methods to predict likelihood of healing of VLUs. Methods that use the area or the shape of the wound rely on the detection of the wound contour, problematic when using imaging due to the two- dimensional nature of images^33^. Further, analysis using the area or shape of the wound is sensitive to rotation or scale due to altered distance or angle between the camera and the wound. This is particularly important when caring for people in their homes because there are many factors that are not within the control of the examiner which would result in weekly differences in the placement of the camera with respect to the wound. Texture analysis provides a set of global features and is insensitive to rotation and scaling factors. Thus, the use of texture analysis is suitable for unsupervised computerised analysis. Another issue when using RGB or thermal images to measure the area of the ulcer is the high likelihood of poor contrast and low resolution of images collected by clinicians, increasing the difficulty of using edge detection to analyse these images. Texture analysis does not require the detection of wound edges^31^. In addition, textural analysis does not require image registration, which increases complexity as these images must then compare shape and size changes of wounds over time. The large number of textural features increases the complexity of image analysis. However, this was overcome by dimensionality reduction and take only the most principal component into account using PCA. This reduction of the amount of data is necessary for analysis makes it suitable for automated analysis.

This study has shown that by using texture analysis, it was possible to predict the likelihood of VLU healing even without needing to control the ambient conditions in which the images were recorded. Ambient thermal conditions, in particular, significantly impact on area analysis of thermal images, preventing accurate and effective prediction of healing. RGB images are impacted significantly by a number of factors such as lighting conditions, temperature of the room and the colour and texture of the individual’s skin^34^. In the current study the images were recorded in the homes of participants with wounds; homecare for wound management is common^15^ and often preferable for older people with wounds^35^, and any diagnostic test needs to be able to function in the environment in which it would most likely to be used. Negating the need for controlling ambient conditions makes this method of analysis highly useful in a wide range of care settings.

Ideally, the likelihood of delayed healing should be detected as early as possible, such as at the first appointment (week 0 of our study). Based on the current study, using week 0 (baseline) data to predict likelihood of healing would only be possible by incorporating additional data that considers the effect of confounding factors, and is adjusted for potential bias. Previous research has suggested using area reduction at two weeks can predict failure to heal at 24 weeks when combined with living alone, higher ulcer severity scores or not treated with high level compression therapy^32^. And yet other research includes factoring in a number of different variables to calculate the potential for delayed healing: components of age (years), ulcer duration (weeks), history of previous deep vein thrombosis in study ulcer leg, living alone, using a mobility aid, slough/necrotic tissue, ulcer area, level of compression, two week reduction in ulcer area and calf circumference^11^. Given this study is considering the ability of textural analysis of the thermal images versus digital planimetry to predict healing, the above-mentioned factors were not considered. Future research will consider their contribution in predicting healing in conjunction with textural analysis of thermal images.

The shape and the area obtained from thermal images based on the temperature distribution is very different from observed using RGB images (Fig. 3(a)) and the shape and surface area obtained using digital planimetry (Fig. 3(b)). This indicates that there are significant differences in the superficial and deeper ulcer conditions and highlights the importance of thermal imaging of the wounds. Further work to understand these differences and their clinical implications is warranted.

An important note is that the above observations were for the pre-wash images, that is, wounds were not cleansed prior to thermal images being taken. We identified that cleansing wounds alters their inherent temperature distribution, thereby rendering any thermal image unsuitable. Current clinical guidelines recommend VLUs are cleansed when dressings and bandages are changed^18^. Therefore, thermal images of wounds for diagnostic purposes need to be taken prior to cleansing of wounds.

One limitation of this study is that the data were collected from an older cohort with a bias towards chronic wounds. Nearly 80% of the wounds were chronic compared with around 30% in other studies^10,11,14,15^. Therefore, while textural analysis is suitable for assessing potential for healing of VLUs in older people, further work is needed that includes younger people with VLUs to ensure generalisability of results to the broader population.

## Methods

### Study design

This was an observational study, where data were collected prospectively from a convenience sample of individuals with VLUs.

### Participants

Clients seen in their homes by a large home health and aged care provider, living in the northern region of metropolitan Melbourne Australia, were selected to be screened for eligibility for the study. Individuals were included if they: were adults, lived in the catchment area of the nursing service, had a VLU diagnosed from clinical indications and either an Ankle Brachial index between 0.8 and 1.2, or a duplex scan indicating no arterial involvement, as per National Guidelines^36^; had sufficient English proficiency to understand the study; had sufficient cognitive ability to understand the study participant information sheet; and were available for consecutive weekly visits. Exclusion criteria were; a non-venous wound primary diagnosis and/or if the individual would heal within the study period, based on wound either almost or fully epithelialized in less than two weeks.

This study was approved by the Human Research and Ethics Committee of Bolton Clarke (Project number: 194) and RMIT University (BSEHAPP 21–15). All participants received a participant information sheet and their informed written consent was obtained. All experiments were performed in accordance with The Declaration of Helsinki (1964) and relevant local and international guidelines and regulations.

### Data collection

Data were collected in participants’ homes by one research nurse with advanced training in wound care. At baseline, the data collected included: demographic data including age, gender, biomedical data including comorbidities; and wound related data including ulcer duration, cause of ulcer, location of ulcer, digital planimetry measurement using Visitrack (Smith & Nephew) from acetate tracing, and digital and thermal images.

Thermal images were collected using ULRIvision TI160 (Zhejiang Ulirvision Technology Co., Ltd). The device is a hand-held imaging device which acquires thermal images with an accuracy of ± 2 °C (Temperature range: -20 °C to + 120 °C) with simple point-and-shoot operation similar to conventional photo cameras. The device provides images with a resolution of 160 × 120 pixels with 25 µm pitch.

Data were acquired across three weekly consecutive time points for analysis. If any one of the three data points was missed, the data could not be included. If this occurred, and the wound had not healed, the participant was recruited as if a new participant. If a participant’s wound healed during data collection, the healed wound area continued to be monitored for five weeks.

**Figure 2:**
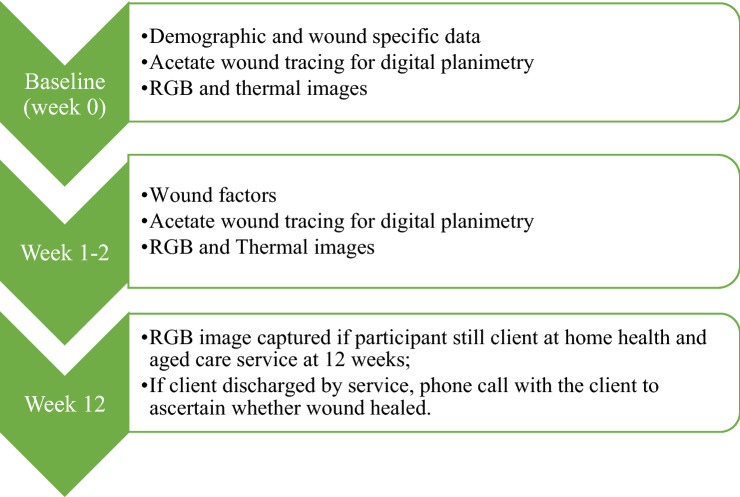
Flowchart of participant data collection.

A wound is classified as healed when it remains epithelialized for a period of two weeks^37,38^. As the wound may still be unhealed (and therefore ‘re-open’) during this time, capturing both digital and thermal images during this period was considered of value to ascertain tissue status. While the thermal images were analysed for the three weeks, RGB images were recorded until week five for all participants, and the healing status of the wounds was monitored until week 12. Participants that had been discharged from the service or were not available for the visits were contacted to ascertain if the wound was still present or healed. See Fig. 2 for a flowchart of the project data collection.

The emissivity parameter for the thermal camera was set to 0.98, corresponding to the emissivity of clean human skin. This parameter is a measure of surface radiation and absorption efficiency, which is required for characterization of human skin temperature using optical devices.

### Digital planimetry

**Figure 3:**
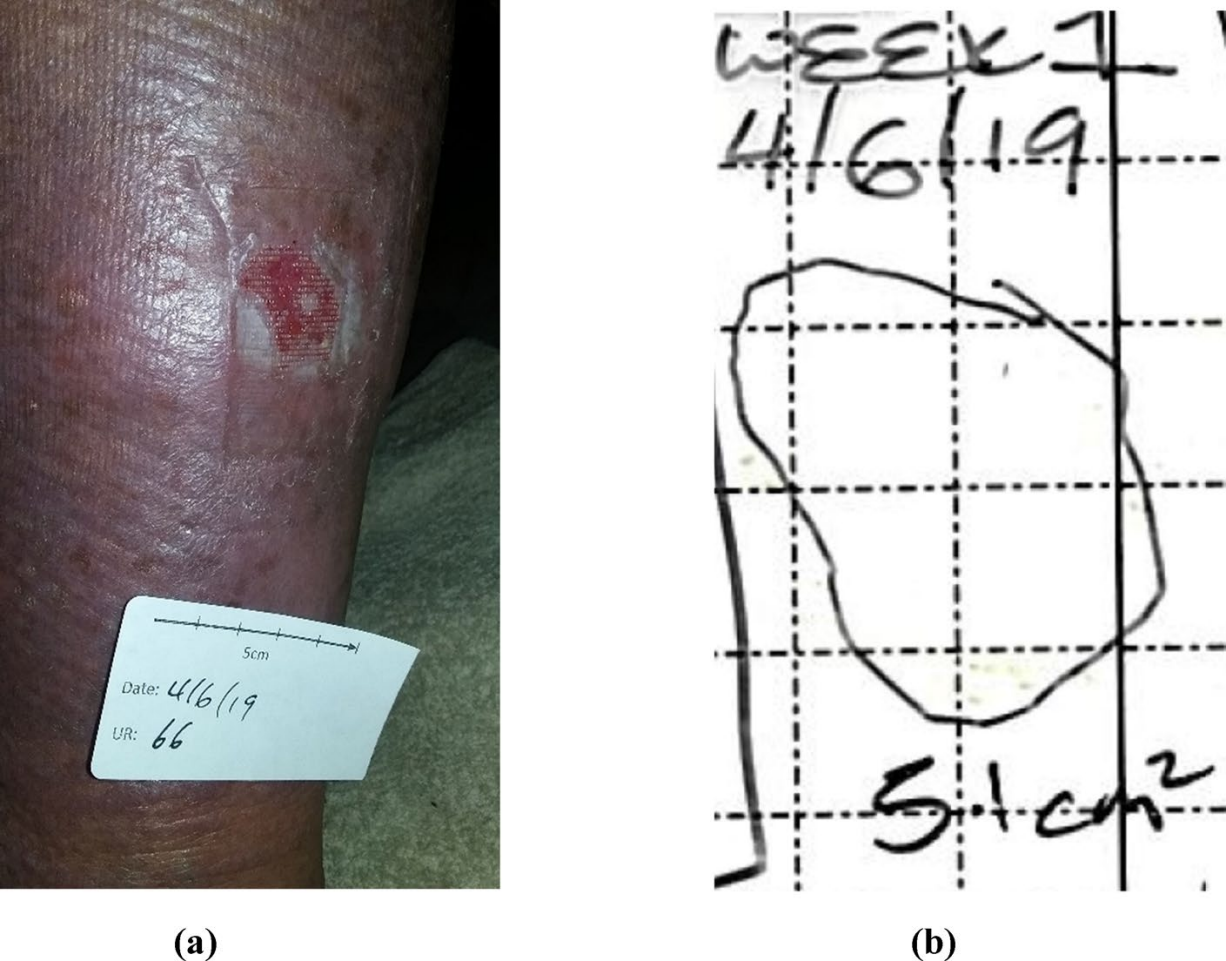
(**a**) An example of digital RGB image showing the ulcer on skin surface (**b**) Wound tracing to obtain the area.

The most widely accepted method to assess ulcers accurately is digital planimetry^18,39,40^. Digital planimetry provides a two-dimensional surface area of ulcers. It involves detecting ulcer margin lines and measuring the inner area by tracing the outline of the ulcer using a marking pen on a clear, sterile, acetate film or graph paper^41^. This tracing is then retraced using a stylus on a portable digital tablet. The area is then calculated using software that counts the number of squares on the film which fall within the ulcer tracing^27^. The areas of the ulcers obtained using digital planimetry, shown in Fig. 3, were used to compare with the thermal image textural analysis.

### Texture analysis

Texture is mostly understood in relation to topographic and textile surfaces. It refers to the surface characteristics and appearance in terms of density, regularity, randomness, uniformity, size, and shape. A texture is often loosely described with terms such as smooth or rough, soft or hard, coarse or fine which provides information about the feel of the surface or its visual impression. For instance, smooth surfaces are even and regular, free from perceptible projections, lumps, or indentation and there is little or no tangible difference between its high and low points. In contrast, rough surfaces are uneven and irregular with a large difference between the high and low points. A similar concept also applies to images, but the texture is characterised by the changes in the brightness value of the pixels and in this case for the thermal images, by the changes in the temperature value of the ulcer and peripheral skin.

In this study the textural properties of the thermal images were studied using the Grey Level Co-occurrence Matrix (GLCM)^42^ which is the distribution of co-occurring temperature values over the thermal images of the ulcer. The GLCM is a statistical method for extracting second-order statistical texture features from an image. It measures the number of times a pixel with value X (i.e. temperature value X) is present adjacent to another pixel with value Y (temperature value Y). Each entry in the GLCM matrix represents the occurrence of pixel value X being found adjacent to the pixel value Y. As the adjacency can be defined to occur in each of four directions (horizontal, vertical, left and right diagonals) in two-dimensional space, four different GLCM matrices can be defined. This method has been successfully used in various applications in medical imaging analysis including the analysis of skin cancer^43^ and detection of macular oedema in retinal images^44^.

The rate of change in textural features, i.e. the ratios of weeks 2 to 1, weeks 2 to 0 and weeks 1 to 0 of same type textural features was calculated for all the texture features. To reduce the dimensionality of such large feature sets (i.e. 12 textural features) while preserving as much information and variability as possible, Principal Component Analysis (PCA) method was used. It transforms the data into a new feature space known as the PCA domain. The first principal component (PCA1) in that domain corresponds to the maximum variance direction in the data, followed by PCA2, as the second largest variance direction which is orthogonal to PCA1. . In this study, it was found that PCA1 and PCA2 were sufficient to model the systematic variation of the dataset.

### Image analysis

The thermal and digital planimetry recordings were analysed using two techniques. First, by looking at the changes in the area of the ulcers across three consecutive treatment weeks using the digital planimetry technique and second by texture analysis of the thermal images of the ulcer. The healing status label of the VLUs included in the analysis was based on the condition of the ulcer at week 12, that is, healed or unhealed.

### Pre-processing and texture analysis

**Figure 4:**
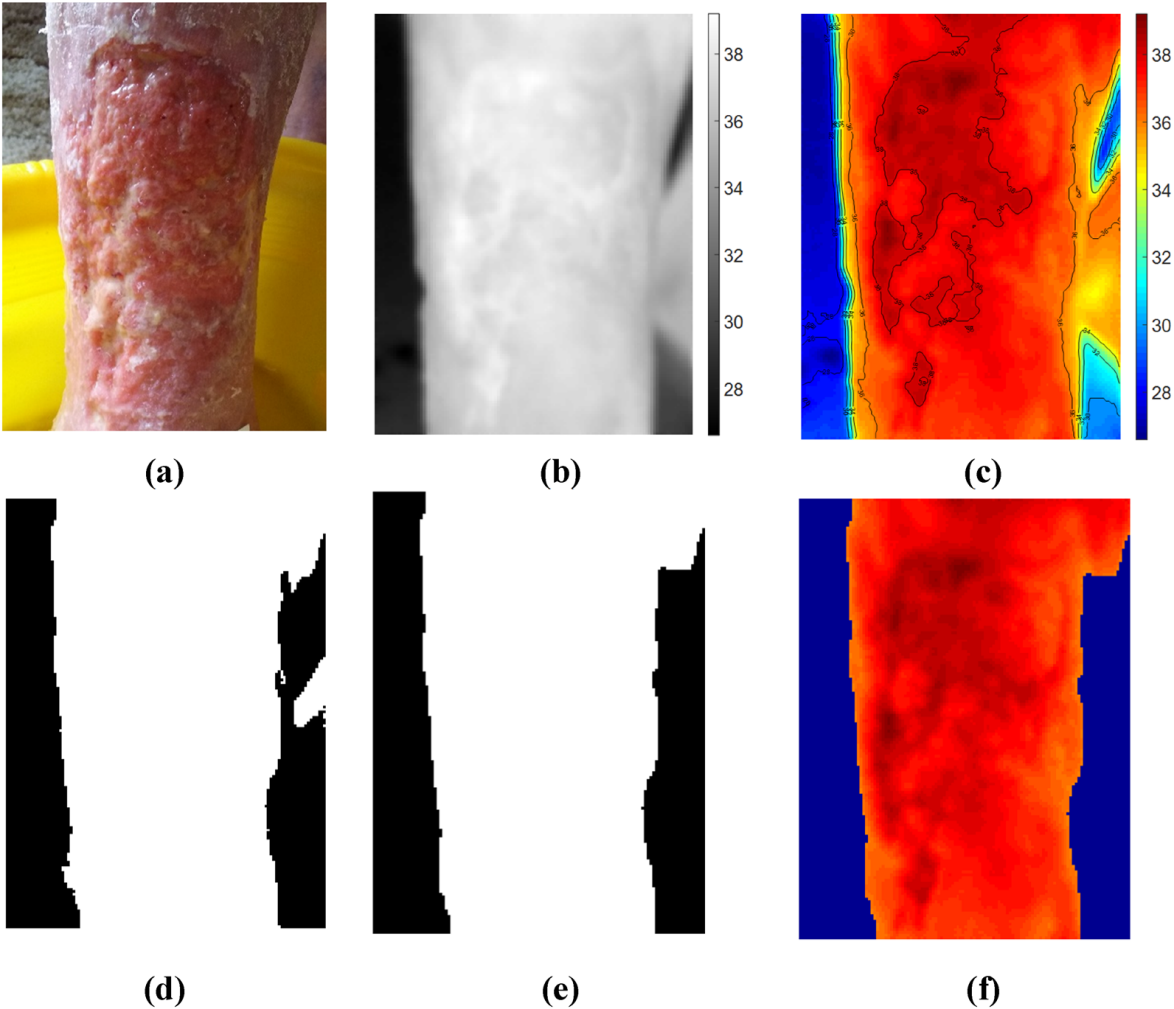
Thermal Image Pre-processing Steps. (**a**) RGB image (**b**) Thermal image normalization (shown in Grey scale) (**c**) Contour detection (**d**) Image masking (**e**) Enhanced mask (**f**) Masked thermal image (background removed).

Thermal image analysis involves the following steps (shown in Fig. 4):

1. Thermal Image normalization
2. Contour detection to differentiate wound bed from background (used for background masking)
3. Creating background mask
4. Morphological operations to enhance the mask
5. Calculation of GLCM matrix
6. GLCM Transformation: Normalization
7. Calculation of textural features: 12 features were calculated from this step:
  - Homogeneity, Angular Second Moment (ASM) (*is a measure of homogeneity of an image*),
  - Contrast (*is measure of contrast or local intensity variation*),
  - Local homogeneity, Inverse Difference Moment (IDM) (*a high IDM value is attributed to homogeneous images),*
  - Entropy (*Inhomogeneous scenes have low first order entropy, while a homogeneous scene has a high entropy*),
  - Correlation (*Correlation is a measure of gray level linear dependence between the pixels at the specified positions relative to each other*),
  - Sum of Squares, Variance (*This feature puts relatively high weights on the elements that differ from the average value*),
  - Sum Average,
  - Sum Entropy,
  - Difference Entropy,
  - Inertia,
  - Cluster Shade, and
  - Cluster Prominence.
8. Calculation of ratios of weeks 2 to 1, weeks 2 to 0 and weeks 1 to 0 of same type textural features (Rate of change in textural features)
9. Principal component analysis (PCA) (First two principal components).

**Figure 5:**
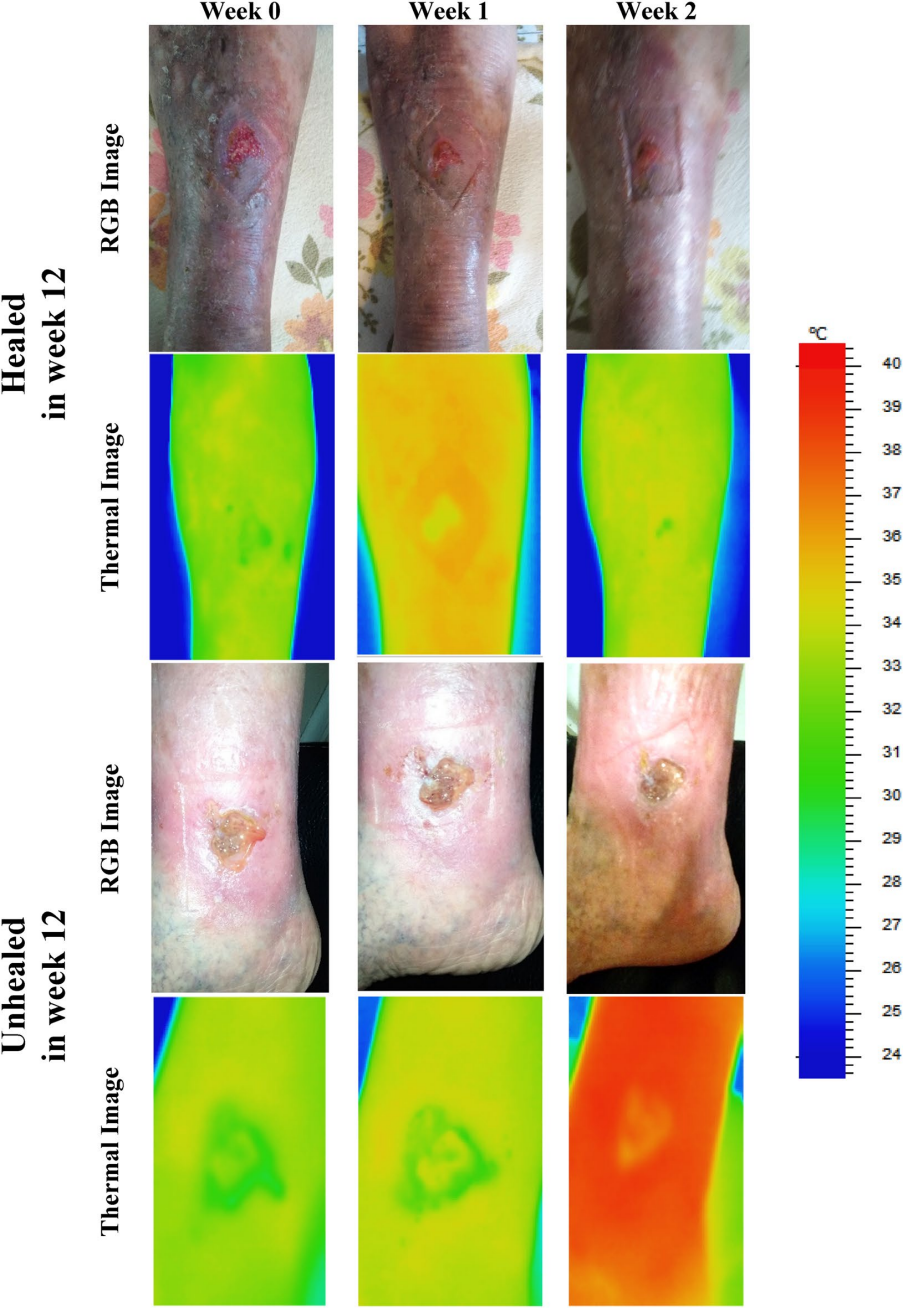
Sample images (RGB and Thermal) of healed and unhealed cases across three consecutive weeks showing the healing progression.

Examples of the final RGB and thermal images used in the analysis across the three weeks in the study are shown in Fig. 5.

### Statistical analysis

All obtained parameters were first tested for normality. With regard to the digital planimetry data, areas of the wounds are different between individual participants, and to measure the change of the area over the weeks, ratios between weeks 2 and 1, weeks 2 and 0 and weeks 1 and 0 were calculated so that data could be pooled. The digital planimetry data did not follow a normal distribution, therefore the non-parametric Kruskal Wallis test was performed to test the difference in ratios between wound areas obtained from digital planimetry across two weeks of ulceration and over three consecutive weeks, grouped according to the healing status of the ulcers at 12 weeks (healed and unhealed).

The interval plots of the area (i.e. ratio data) with 95% confidence interval were also obtained to study the distribution of the area in the healed and unhealed cases. Descriptive statistics, including mean, median, standard deviation (SD) of two-week ratios of area were also computed for healed and unhealed wounds.

To measure the change of the area over the weeks, ratios of weeks 2 to 1, weeks 2 to 0 and weeks 1 to 0 of same type textural features were calculated and then PCA was taken to reduce the dimensionality and pick the first two principal components. Analysis of Variance (ANOVA) was performed to test the group difference between the healing and unhealed ulcers. This established the relationship between the change in textural features in week 2 with respect to the baseline (i.e. week 0) with reduced dimensionality (i.e. First and second principal component, (PCA 1 and PCA 2) and healing status of the VLUs).

All statistical analyses were performed in Minitab (v.16.1) Statistical Software^45^, (www.minitab.com).

### Ethics

All experiments and data collection were performed in accordance with Helsinki accordance of Human experiments, and relevant local and international guidelines. All recordings were after obtaining signed informed consent from the participants. The details of the ethics board approval are provided in the Methods section.
